# A phase I study of an agonist anti-CD27 human antibody (CDX-1127) in patients with advanced hematologic malignancies or solid tumors

**DOI:** 10.1186/2051-1426-1-S1-P127

**Published:** 2013-11-07

**Authors:** Howard Burris, Stephen Ansell, John Neumanitis, Geoffrey Weiss, Branimir Sikic, Donald Northfelt, Lana Pilja, Thomas Davis, Michael Yellin, Tibor Keler, Timothy Bullock

**Affiliations:** 1 Sarah Cannon Research Institute, Nashville, TN, USA; 2 Mayo Clinic, Rochester, MN, USA; 3 Mary Crowley Cancer Research Center, Dallas, TX, USA; 4 University of Virginia, Charlottesville, VA, USA; 5 Stanford Cancer Institute, Stanford, CA, USA; 6 Mayo Clinic, Scottsdale, AZ, USA; 7 Celldex Therapeutics, Inc., Phillipsburg, NJ, USA

## 

CD27, a member of the tumor necrosis factor receptor superfamily, is constitutively expressed on the majority of mature T cells, memory B cells, and a portion of NK cells. Previously we have reported anti-tumor and immunological properties of the fully human anti-CD27 mAb 1F5 (CDX-1127) in murine tumor models of solid and hematologic tumors. A Phase I dose escalation study of CDX-1127 in patients (pts) with recurrent or treatment-refractory B-cell malignancies and specific solid tumors is ongoing. CDX-1127 (0.1 to 10 mg/kg IV) was administered as a single-dose with 28-day observation, followed by 4 weekly doses before restaging at Day 85. Up to 4 additional re-treatment cycles (consisting of 4 weekly doses) were permitted in pts with stable disease or tumor response. Dose-escalation in pts with solid tumors has completed with preliminary results as follows: 25 pts (10 colorectal, 7 melanoma, 3 ovarian, 2 prostate, 2 renal cell, and 1 NSCLC) received 0.1, 0.3, 1, 3 or 10 mg/kg CDX-1127, with one DLT (Grade 3 hyponatremia) at 1 mg/kg. Treatment-related toxicity, generally Grade 1-2, included fatigue, chills, hyperhydrosis, decreased appetite, rash and diarrhea. Flow cytometry and functional immune analysis of peripheral blood lymphocytes from these pts showed evidence of immunomodulatory activity, including increase in expression of activation markers on T cells (HLA-DR), increases in NK cells, decrease in Tregs, and enhanced T cell response to various stimuli in in vitro assays. Of the 18 pts that completed at least one cycle of therapy, 4 pts had stable disease (range: 2.4+ to 11+ months), including a renal cell carcinoma pt that has completed 5 cycles of therapy. To date, no pts have achieved formal objective response by RECIST. One pt with colorectal cancer had 33% shrinkage of measurable disease by RECIST but this was associated with new lesions; by irResponse criteria (Wolchok 2009) the patient had irSD with 45% shrinkage in measurable lesions. This study shows that weekly dosing of CDX-1127 is well tolerated with biologic activity. Additional tumor-specific expansion cohorts (including melanoma and renal cell carcinoma) are being enrolled to further define the biological and clinical activity of CDX-1127 as a single-agent.

